# Supplementary material for: The effectiveness of the buddy program training module to enhance the daily living function, social participation and emotional status of older adults in residential aged care homes
Source: PLoS One. 2024 Apr 3;19(4):e0301544. doi: 10.1371/journal.pone.0301544 (PMC10990167; doi:10.1371/journal.pone.0301544)
Supplement: Attachment — Submitted filename: Response to Reviewers.docx.pdf [file pone.0301544.s001.pdf]

Response to Reviewers

Journal requirements:

1. When submitting your revision, we need you to address these additional requirements.

Please ensure that your manuscript meets PLOS ONE's style requirements, including those for file naming. ThePLOS ONE style templates can be found at https://journals.plos.org/plosone/s/file?id=wjVg/PLOSOne_formatting_sample_main_body.pdf and https://journals.plos.org/plosone/s/file?id=ba62/PLOSOne_formatting_sample_title_authors_affiliations.pdf.

The manuscript was revised and corrected based on the PLOS ONE's style requirements

2. In your Data Availability statement, you have not specified where the minimal data set underlying the results described in your manuscript can be found. PLOS defines a study's minimal data set as the underlying data used to reach the conclusions drawn in the manuscript and any additional data required to replicate the reported findings in their entirety. All PLOS journals require that the minimal data set be made fully available. For more information about our data policy, please see http://journals.plos.org/plosone/s/data-availability.

""Upon re-submitting your revised manuscript, please upload your study's minimal underlying data set as either supporting Information files or to a stable, public repository and include the relevant URLs, DOIs, or accession numbers within your revised cover letter. For a list of acceptable repositories, please see http://journals.plos.org/plosone/s/data-availability#loc-recommended-repositories. Any potentially identifying patient information must be fully anonymized.

Important: If there are ethical or legal restrictions to sharing your data publicly, please explain these restrictions in detail. Please see our guidelines for more information on what we consider unacceptable restrictions to publicly sharing data: http://journals.plos.org/plosone/s/data-availability#loc-unacceptable-data-access-restrictions. Note that it is not acceptable for the authors to be the sole named individuals responsible for ensuring data access.

We will update your Data Availability statement to reflect the information you provide in your cover letter.

Data cannot be shared publicly because of confidentiality. Data are available from the Ethics Committee (contact via sepukm@ukm.edu.my) for researchers who meet the criteria for access to confidential data.

3. Please include your full ethics statement in the 'Methods' section of your manuscript file. In your statement, please include the full name of the IRB or ethics committee who approved or waived your study, as well as whether or not you obtained informed written or verbal

consent. If consent was waived for your study, please include this information in your statement as well.

The ethics statement with the full name of the IRB or ethics committee was added in the 'Methods' section (Line 128-129) The information regarding informed consent was added under the 'Study Procedure' (Line 244-248)

4. Please review your reference list to ensure that it is complete and correct. If you have cited papers that have been retracted, please include the rationale for doing so in the manuscript text, or remove these references and replace them with relevant current references. Any changes to the reference list should be mentioned in the rebuttal letter that accompanies your revised manuscript. If you need to cite a retracted article, indicate the article's retracted status in the References list and also include a citation and full reference for the retraction notice.

Reference lists were checked and no reference was removed

Reviewer #1

1) The authors did a work worth publishing to an international audience and did a rigorous modern statistical analysis. However, questions about the methodology. The control group that were selected was based on a mini mental state of 19 and above, which supposes that they had mild to normal range of cognition.

   The involvement of the participants in the control group that was selected based on the mental state of 19 and above was referred to the previous study done by Yang et al. (2016). Most of the participants in the control group attended primary school in this study was 80% and according to the result of the mini mental state, most of the participants were unable to perform tasks related to paper and pencil, but it is deemed sufficient to be included in this study.

2) Also. the method of recruiting the participants presupposes adequate intellectual functioning and ability to read in Malay and understanding, especially among the participants. Adverts on the notice board was the major method of information which means that they were voluntary, but questions remain about understanding of information presented and attention paid by the elderly to these notices in a rehab. If cognitive functioning is poor then understanding and informed consent may be doubtful as kins were not contacted.

   For illiterate participants, the information about the research was explained by researchers until they fully understood the purpose and their involvement in the study and the were given sufficient time to understand the aim of the study and they could ask any questions regarding the study and once they agree to take part in the study, the consent form was given. Also, as they reside in the residential aged care homes, impartial witness was present to complete the consent process.

3) Additionally, the authors included all races but limited the restriction to verbally and readability in Malay language , only to suggest translations as recommendation ?

The new module is developed in the Malay language because in Malaysia regardless of race, the Malay language is the national language and is generally understood by people of all races. The suggestion to translate to other languages could encourage the use of the module to focus on a specific group of races and could provide the psychometric properties of the module through the effectiveness of the module in different language

4) Why were 2 out of 10 chosen randomly, that is 0.2, within the sub region of similar social setting .i feel it was rather a convenience sample. The outlay of the residential groups should have been structured in such a way to represent more generalization.

The simple randomization was chosen in this study to eliminate selection bias.

5) The confounding factors are much and authors should note that even age and worsening dementia along with age may be a factor for differences in BADL.

The confounding factors may be a factor for differences in BADL and worth investigating, however, in this study, the daily living function, social participation, and emotional status were chosen to identify the effectiveness of the newly developed buddy program training module.

6) The quasi experimental was skewed to more of Malay residents and little representation of other groups and religion. How did they translate the instruments was there an effort to back translate in order to achieve more appropriate content validity and reliability.

Malay represents the majority of the ethnicity in Malaysia. Also, for the government residential aged care homes, the majority of the residents were Malay residents. The translation of the future instrument may focus on the specific races in the specific centres according to their own language such as Chinese or Tamil, to investigate the validity and reliability of the translated version.

Reviewer #2: Reviewer's comments (Yetunde Adeniyi)

Overall, it is a good paper that should be considered for publication. It emphasizes community participation and peer support.

1) Under the inclusion criteria, the cut off mark on MMSE for both the guiding adults and guided adults were put at scores above 19. Authors should attend to this, the cut off points for both groups should not be the same

In the submitted manuscript, we have identified a typographical error for the word 'above' that requires correction. The inclusion criteria for older adults as 'guided

friends, were residents who (iii) have impaired cognitive functioning [Mini-Mental State Examination (MMSE) score below 19 marks] (line 165-167)

2) The authors should provide a summary of the study procedure, what we have in the paper right now are detailed description of the study instruments. There is a need to have a separate section for study procedures detailing what was done.

The study procedure was included previously under the Participant section. The information about the summary of the study procedure has been moved after the Instrument section as suggested (line 240-247)
